# Four Weeks of a Neuro-Meditation Program Improves Sleep Quality and Reduces Hypertension in Nursing Staff During the COVID-19 Pandemic: A Parallel Randomized Controlled Trial

**DOI:** 10.3389/fpsyg.2022.854474

**Published:** 2022-05-11

**Authors:** Christophe Hausswirth, Xavier Nesi, Alexandre Dubois, François Duforez, Yann Rougier, Katie Slattery

**Affiliations:** ^1^LAMHESS, University of Côte d’Azur, Nice, France; ^2^BeScored Institute, Sophia Antipolis, France; ^3^School of Sport, Exercise and Rehabilitation, University of Technology, Sydney, NSW, Australia; ^4^Hotel-Dieu de Paris, Centre du Sommeil et de la Vigilance, Paris, France; ^5^WHealth Foundation, Villeneuve-Loubet, France

**Keywords:** sleep, neuro-meditation, synchromotherapy, heart rate variability, hypertension

## Abstract

The purpose of this study was to examine the effectiveness of a neuro-meditation program to support nurses during the COVID-19 pandemic. Forty-five (10 men and 35 women) nurses were classified into three groups based on their systolic blood pressure: normotensive (G-nor; *n* = 16, 43.8 ± 11.0 year), hypertensive (G-hyp; *n* = 13, 45.2 ± 10.7 year) and control (G-con; *n* = 16, 44.9 ± 10.6 year). Using a parallel, randomly controlled design across a 4-week period, 10 × 30-min sessions using the Rebalance© Impulse were completed. Sleep was assessed by wrist actigraphy and subjective sleep questionnaires; perceived sleep quality, Ford Insomnia Response to Stress Test questionnaire and the Spiegel Sleep Quality questionnaire (SSQ). Blood pressure, resting heart rate, mean heart rate (HRmean), heart rate variability index (RMSSD), cortisol, and alpha-amylase were also measured. Statistical analysis was completed using factorial ANOVA. Sleep improved in the G-hyp group; SSQ (*p* < 0.01); perceived sleep quality (*p* < 0.01); sleep efficiency and fragmentation index (*p* < 0.05). In the G-nor group, sleep was improved to a lesser extent; perceived sleep quality (*p* < 0.01). A significant time–group interaction was reported in resting heart rate (*p* < 0.01), systolic blood pressure (*p* < 0.01), and diastolic blood pressure (*p* < 0.05) with these measures being significantly reduced in the G-hyp group. RMSSD increased in the G-nor group (*p* < 0.01). This initial evidence suggests that neuro-meditation reduces excessive sympathetic activity, promoting enhanced sleep quality and autonomic control during periods of increased work-related stress.

**Clinical Trial Registration:**

The study was conducted at Bioesterel, Sophia-Antipolis, France as a clinical trial: Neuro-meditation improves sleep quality, https://www.drks.de/ui_data_web/DrksUI.html?locale=en, DRKS00025731.

## Introduction

Work-related stress and burnout are a common occurrence in health care employees (Monsalve-Reyes et al., 2018). Nurses experience both physical and psychosocial stress as part of their role (Arora et al., 2010). When prolonged, or intensified, this stress may manifest as symptoms, such as insomnia, headaches, depression, anxiety, fatigue, increased blood pressure, autonomic disfunction, or muscle and joint pain (Milliken et al., 2007). Moreover, nurses working under stressful conditions may have an increased error rate, make poor clinical decisions, and be slower to complete tasks (Arora et al., 2010). This not only affects the safety of the patients, but also increases the risk of burnout and the likelihood of developing chronic diseases in nursing staff (Keane et al., 1985).

The intense workload, uncertainty, and lack of resources during the COVID-19 (severe acute respiratory syndrome coronavirus 2) pandemic has placed all health care workers under extremely stressful conditions and at an increased risk of burnout (Galanis et al., 2021). A study by Badahdah et al. (2020) revealed that, of the 509 doctors and nurses assessed, 25.9% had severe anxiety and 56.4% experienced extreme stress during the initial wave of the pandemic. Similarly, in a cross-sectional study of 1,257 frontline health care workers treating COVID-19 patients in Wuhan, the rate of depression, anxiety, insomnia, and distress were 50.4%, 44.6%, 34.0%, and 71.5%, respectively (Lai et al., 2020). Of particular concern is the high prevalence of insomnia (+38.9%) reported in a recent meta-analysis investigating the impact of COVID-19 on nurses and doctors (Pappa et al., 2020). As the pandemic continues, it is important to provide support and targeted interventions for health care workers to manage work-related stress and promote their physical and mental wellbeing.

Mindfulness-based interventions (MBI) may be an effective strategy to improve sleep quality and reduce the impact of stress-related symptoms (Creswell, 2017; van der Riet et al., 2018; Rusch et al., 2019). There is building evidence to suggest that MBI can affect the activation of the sympathetic nervous system and help regulate stress reactivity (Pascoe et al., 2017). As the maintenance of attention and self-control required with meditative practice can strengthen positive-cognitive emotional processes (Amutio et al., 2015). When exposed to a stressor, it has been proposed, that this enhanced emotional control reduces the adverse physiological reactions that would typically occur (Heckenberg et al., 2018). This has been demonstrated by improved sleep quality (Rusch et al., 2019); a decrease in resting blood pressure (Creswell et al., 2009; Pascoe et al., 2017); cortisol (Creswell et al., 2009), and resting heart rate (Pascoe et al., 2017) following MBI. These findings, while not always observed (Pascoe et al., 2017), warrant further investigation.

As with any skill, it can be difficult for those who are inexperienced with meditation to develop the level of awareness and attention required to significantly benefit from the practice. By providing electroencephalogram biofeedback, neuro-meditation may speed the learning process and allow individuals to achieve and maintain the desired state of consciousness more quickly, thereby increasing the effectiveness of the program (Brandmeyer and Delorme, 2013; Tarrant, 2020). The purpose of this study was to evaluate the influence of a MBI on sleep and other stress-related parameters in nurses under increased work-related stress due to the COVID-19 pandemic. We hypothesize that a four-week MBI using neuro-meditation will improve the sleep quality of all participants and normalize stress-related symptoms. We also anticipate that the use of coach-guided exercises combined with light simulation will accelerate the participant’s ability to engage in mindful meditation, resulting in the relatively short four-week program leading to comparable benefits that are typically only observed after longer (~2–3 months) MBI.

## Materials and Methods

### Participants

A convenience sample of 45 people (10 men and 35 women) aged 25–61 working as nursing staff in hospitals and assisted living facilities in the region were recruited. After being fully informed of the purpose and protocols, all participants gave informed consent. The study was approved by the University of Technology, Sydney (ETH21-6116) in accordance with the Declaration of Helsinki (1964; revised 2001) and registered with the German Clinical Trial Register (DRKS00025731). It conformed to the Committee on Publication Ethics (COPE) and the International Committee of Medical Journal Editors (ICMJE) recommendations. The investigation was conducted from June to August 2020 to examine strategies that can reduce stress and improve the health and wellbeing of nursing staff under extreme workloads during the initial wave of the COVID-19 pandemic. Immediately prior to the investigation, France was locked down in a state of health emergency from March to May 2020 with a peak in COVID-19-related visits to emergency departments (*n* = 5,853) and SOS Médecins (*n* = 1,777) occurring on the 27 March 2020 (Thiam et al., 2022).

During the initial visit to the laboratory (Bioesterel, Sophia Antipolis, France), each participant was examined by a cardiologist and a medical doctor. Participants were excluded if they showed premature heart beats, serious abnormal heart rhythms, suffered from muscular and/or joint disorders, were on hypertensive, antidepressant, psychotropic, or anxiolytic medication. Those who reported a current inflammatory disorder, sleep apnea, restless legs syndrome, autoimmune disease, type 1 diabetes mellitus, hepatitis C, cancer, or acute infection in the past 2 weeks were excluded. Participants that reported sleep apnea or restless legs syndrome were excluded as these conditions can influence sleep measures but are not related to sleep. Also excluded were people with narcolepsy, epilepsy, central disorders of hypersomnolence, irregular sleep–wake rhythm disorder, and parasomnia. These pathologies are known to influence actimetry and polysomnography values (Alakuijala et al., 2021). Systolic blood pressure (SBP), diastolic blood pressure (DBP), and resting heart rate were measured seated by electrosphygmomanometry (Tango+; Suntech Medical, Flaxlanden, France). Participants were then classified into three groups based on their SBP (Table 1): Hypertensive (G-hyp), for those with a SBP higher than 140 mmHg (stage 2 hypertension; Muntner et al., 2019). Basic randomization using the flip of a coin was then used to allocate the remaining participants into either the normotensive (G-nor) or control (G-con) group (Altman and Bland, 1999). There were no significant differences between the three groups in terms of body composition or anthropometric measures (Table 1).

**Table 1:** Participant gender, age, and body composition (mean ± SD).

Characteristic	G-con (*n* = 16)	G-nor (*n* = 16)	G-hyp (*n* = 13)
Gender (M/F)	5/11	2/14	3/10
Age (years)	44.9 ± 10.6	43.8 ± 11.0	45.2 ± 10.7
Body mass index (kg/m^2^)	26.1 ± 5.6	25.6 ± 5.8	27.2 ± 5.3
Fat mass (%)	30.1 ± 8.2	30.5 ± 8.4	32.0 ± 7.6

G-con, control group; G-nor, normotensive group; G-hyp, hypotensive group; M, male; and F, female.

### Study Design

**Figure 1:**
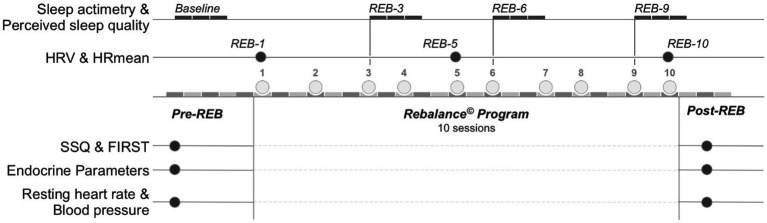
Experimental design. Baseline, 3-day data collection period prior to Rebalance© session 1; REB-1 −5, −10, Rebalance© session number 1, 5, and 10, respectively; REB-3 −6, −9, 3-day data collection period following Rebalance© session number 3, 6, and 9, respectively; Pre-REB: prior to Rebalance© Program; Post-REB: following Rebalance© Program. HRV, heart rate variability; HRmean, average heart rate during HRV measurement; SSQ, Spiegel Sleep Quality questionnaire; FIRST, Ford Insomnia Response to Stress Test.

A parallel randomized control trial was used to establish the effectiveness of a MBI using the Rebalance© Impulse (Rebalance Tech Corp., Miami, United States). Figure 1 provides an overview of the experimental design. Resting blood pressure, resting heart rate, the Ford Insomnia Response to Stress Test questionnaire (Drake et al., 2006; FIRST), the Spiegel Sleep Quality questionnaire (SSQ; Klimm et al., 1987), body composition (body weight, percentage of body fat), anthropometric measures and blood tests were completed in the days preceeding the first MBI session (Pre-REB). This same battery of tests was completed 48 h after the last session (Post-REB) to determine the changes made by the 10-session Rebalance© Program. Participants completed a 12 h fast prior to body composition assessment with a bio-electrical impedance analysis device (Tanita MC780 MA model; Tanita Europe BV, Amsterdam, Netherlands), validated in comparison with the dual-energy X-ray absorptiometry (DEXA; fat mass (kg) ICC = 0.88; *r* = 0.89; Verney et al., 2016). Mean heart rate (HRmean) and heart rate variability (HRV) were measured at three distinct time points: Prior to the first Rebalance session (REB-1), the fifth session (REB-5), and the tenth session (REB-10) in the G-nor and G-hyp groups. HRmean and HRV were measured in the G-con in a supine position at similar time points during their first visit to the laboratory and again 2 and 4 weeks later.

### Study Procedures

#### The Rebalance© Program

**Figure 2:**
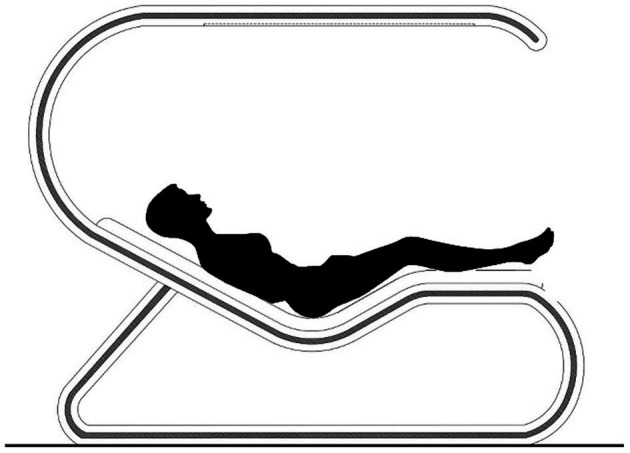
Schematic representation of the Rebalance© Impulse.

The G-nor and G-hyp groups followed the same Rebalance© Program during the four-week intervention where 2–3 sessions were completed per week. Participants completed their session individually at a similar time of day on each occasion in the Rebalance© Impulse room at the laboratory in Bioesterel, Sophia Antipolis, France. The G-con group did not receive any treatment. Rebalance© Impulse is a non-invasive cognitive stimulation and mindfulness training device based on applied neuroscience (Creswell, 2017; Cheron et al., 2022). During the session, participants lied down in a “zero gravity” position (Figure 2). The mindfulness training included sound therapy and coach-guided meditation associated with light stimulations (synchromotherapy). This device has already been the subject of a scientific study (Cheron et al., 2022). The software adapted the frequency of light stimulations based on the real-time reading of the dominant wave at the beginning of the Rebalance© Impulse session to bring the brain biorhythm into the so-called alpha zone (between 8 and 13 Hz). More specifically, five frequency levels were used in real time, allowing for an observed dominant wave to evolve gradually (3–4 min) toward the target brain biorhythm, namely, the dominant range theta (between 4 and 7 Hz) and the alpha waves. One of the main objectives of the Rebalance© Impulse programs was to restore a balance to the waking state between the alpha brain waves and the beta waves (between 13 and 30 Hz) by slowing down the latter, whose predominance leads to exhaustion (Craig et al., 2012). The light stimulations were created using a disk composed of LED bars placed at regular intervals on the rays of the disk. The user perceived a dynamic circle with an increasing or decreasing diameter when the LEDs switched on and off at a predefined rate one after the other from the beginning of the ray (center of the disk) to the end of the ray (edge of the disk). The Rebalance© Impulse ceiling included a circle of 16 radial arms each one including an array of LEDs providing continuous or intermittent rhythmic waves presenting eight different color lightings ranging from purple (415 nm) to red (720 nm) with different levels of illuminance ranging from 7 to 120 lx. The software also created the OBF™ (Optimal Brain Flow) Index based on the brain wave measurements recorded throughout the Rebalance© Impulse session. It primarily considered the alpha wave and the theta wave (between 4 and 7 Hz), both in terms of power and duration during the session. This OBF™ Performance Index characterized the state of neuro-meditation of each session. In addition, the animation and choice of color of the LEDs were programmed to match the voice-guided exercises. Each session was 30 min in duration.

#### Sleep Actigraphy

Sleep was monitored across four, 3-day periods at Baseline (three nights prior to the Rebalance© Program), and for three nights following the third (REB-3), sixth (REB-6), and ninth (REB-9) Rebalance© session. These time points were chosen to ensure an appropriate baseline prior to commencement of the MBI (Hausswirth et al., 2014; Schaal et al., 2015), track time course changes and to capture the cumulative effect of the MBI following the intervention. Participants wore an Actiwatch sleep monitor (Cambridge Neurotechnology Ltd., Cambridge, United Kingdom) on the non-dominant wrist with the epoch length set to 1 min. The watch was worn at all times of the day (other than when the watch could get wet) for each monitoring period. Mean behavioral activity over each recording period was automatically calculated using the Sleepwatch Sleep Analysis software (version 5.28, Cambridge Neurotechnology Ltd.).

During each monitoring period, participants completed daily sleep diaries to ensure reliable sleep–wake scoring (Fietze et al., 2009). While completing the actigraphy, perceived sleep quality was also recorded on a seven-point scale (from very, very good to very, very poor) upon waking each morning. The following dependent variables were derived from the sleep diary and activity monitor data:

- Time in bed (h:min): the amount of time spent in bed attempting to sleep between bedtime and get-up time.
- Bedtime (hh:mm): the self-reported clock time at which a participant went to bed to attempt to sleep.
- Get-up time (hh:mm): the self-reported clock time at which a participant got out of bed and stopped attempting to sleep.
- Sleep onset latency (min): the period between bedtime and sleep start.
- Actual sleep time (h:min): the time asleep from sleep start to sleep end.
- Sleep efficiency (%): sleep duration expressed as a percentage of time in bed.
- Fragmentation index: a measure of restlessness during sleep, using the percentage of epochs where activity is >0.
- Immobile time (min): the actual time spent immobile during time in bed.

#### Sleep Instruments

The SSQ (Spiegel, 1981; Klimm et al., 1987) is a validated questionnaire commonly used in French clinical sleep settings and epidemiology to provide a quantitative and qualitative assessment of a patient’s perceived quality of sleep (Sadeh, 2015). The SSQ is comprised of six questions. The maximum score is 30, and impaired sleep is defined as a score < 24; a pathological sleep pattern exists if the score is <15. Sleep reactivity is a key variable and provides early indication that a participant is at risk of developing a sleep disorder. Sleep reactivity was measured using FIRST (Drake et al., 2006) that determines the impact of a participant’s sleep, represented through the qualitative modifications of sleep according to situations experienced in daily life. The FIRST is a nine-item scale used to assess an individual’s likelihood of experiencing sleep difficulties in response to common stressful situations. Each item is self-rated on a four-point Likert scale and summed to yield a total score (range: 9–36); higher scores indicate higher levels of sleep reactivity.

#### Heart Rate

Heart rate data (OH1, Polar, Kempele, Finland) was collected in R–R interval mode for 4 min while the participant was lying quietly in the dedicated Rebalance© Impulse room. Data from the last 3 min of each sampling period were used for analysis, to allow the heart rate to stabilize. No particular breathing frequency was imposed (Saboul et al., 2013). For all HRV samples, it was subsequently verified that the respiration rate was always in the high-frequency range (0.15–0.50 Hz).

HRV data were analyzed using specialized HRV analysis software (Kubios HRV analysis Software, Finland; Tarvainen et al., 2014). Data were processed by the same individual and visually inspected to identify artifacts and occasional ectopic beats, which were removed manually. The time-varying HRV indices kept for analysis were the root mean square difference of successive normal R–R intervals (RMSSD). The mean heart rate (HRmean) of the 3 min period was also recorded.

#### Endocrine Analysis

Blood was drawn and collected into one 6-ml serum tube and one 6-ml EDTA plasma tube (Becton, Dickinson and Company Vacutainer, Franklin Lakes, NJ). These were centrifuged at 3,000 *g* for 10 min and promptly frozen at −80°C. The samples were obtained between 7:00 AM and 8:00 AM. Standard enzyme-linked immunoassay procedures were used to determine cortisol (IBL International; Toronto, ON, Canada) and alpha-amylase concentrations (α-amylase; IBL International). All measures were performed in duplicate with intra-assay coefficients of variation of 10% or less.

### Statistical Analysis

All data were stored in an electronic database and analyzed using specialized statistical software (SPSS v20.0, Chicago, IL, United States). Results are expressed as mean ± standard deviation (SD). The normality of distribution for each variable was tested using the Shapiro–Wilk test. Statistical analysis was completed using a factorial ANOVA by group (G-con, G-nor, and G-hyp) and time (Pre-REB, Post-REB) for SBP, DBP, resting heart rate, SSQ, FIRST, and endocrine variables; and time (Baseline, REB-3, REB-6, and REB-9) for actimetry parameters; and time (REB-1, REB-5, and REB-10) for HRV and HRmean variables. If a significant time–group interaction effect was observed, Tukey’s Honest Significant Difference tests were performed as post-hoc analysis to further discern differences. When assumptions of normality or homogeneity of variances were not met, the data were log-transformed before analysis. Means were then de-transformed back to their original units. The criteria to interpret the magnitude of effect size was >0.2 *small*, >0.5 *moderate*, >0.8 *large*, and >1.3 *very large* (Cohen, 1988). An *a priori* sample analysis revealed that 10 pairs of subjects were the minimum required in a matched pair design to be able to reject the null hypothesis that this response difference is zero with probability (power) 0.8. The Type I error probability associated with the test of this null hypothesis is 0.05 (G*Power version 3.1.3, Universitӓt Kiel, Germany). Statistical significance was accepted at *p* < 0.05.

## Results

### Sleep Analysis

**Figure 3:**
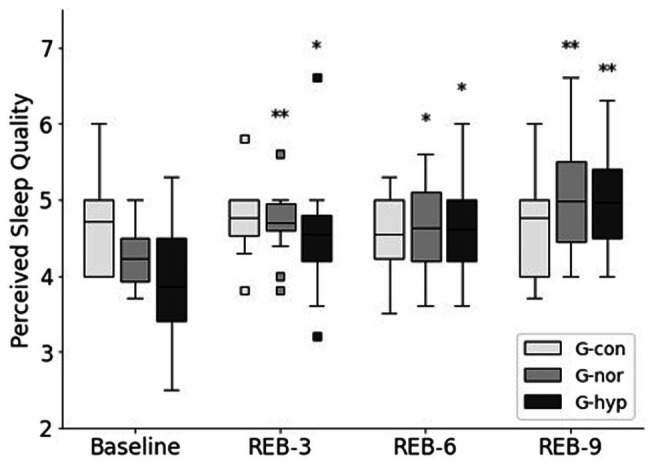
Changes in perceived sleep quality throughout the Rebalance© Program. G-con, control group; G-nor, normotensive group; G-hyp, hypotensive group. Baseline, 3-day data collection period prior to Rebalance© session 1; REB-3, −6, −9, 3-day data collection period following Rebalance© session number 3, 6, and 9, respectively. Box plots represent median interquartile range (IQR, Q25–Q75), and error bars are maximal and minimal observations within 1.5 × IQR. Squares represent maximum and minimum observations above or below 1.5 × IQR. * and **Significantly different from Baseline (*p* < 0.05 and *p* < 0.01, respectively).

All participants completed the entire study protocol. Calculated scores for the SSQ and FIRST are presented in Table 2. A significant time–group interaction was reported in SSQ (*p* < 0.01) and FIRST (*p* < 0.05). In G-con, no significant change was reported between the Pre-REB and Post-REB periods. Sleep quality measured by the SSQ was significantly improved in the G-hyp group (*p* < 0.05 after the Rebalance© Program. Sleep quality also improved in the G-nor group; however, the difference did not reach significance (*p* = 0.061). Sleep reactivity did not change in response to the Rebalance© Program. However, the FIRST score at Post-REB approached significance for the G-hyp group compared to Pre-REB (*p* = 0.062). For the G-nor and G-hyp groups, the perceived sleep quality was significantly increased between the Baseline and REB-3 and remained unchanged until the end of the Rebalance© Program (Figure 3). The overall improvement in perceived sleep quality was 19.1% ± 14.2% and 28.2% ± 18.6% for the G-nor and G-hyp groups, respectively (both *p* < 0.01), with no significant change for G-con (2.3% ± 12.8%; *p* = 0.87). Raw values of sleep actigraphy parameters for the three groups are presented in Table 3. There was no significant time–group interaction in time in bed (*p* = 0.90), assumed sleep time (*p* = 0.79), actual sleep time (*p* = 0.97), and immobile time (*p* = 0.42). A significant time–group interaction effect was observed for fragmentation index and sleep efficiency (both *p* < 0.05). For all parameters, no significant change was reported for the G-con group during the Rebalance© Program. For the G-hyp group, sleep efficiency and fragmentation index were significantly improved at the end of the program, whereas only a significant increase in sleep efficiency was observed in the G-nor group from the ninth session.

**Table 2:** Changes in sleep quality, sleep reactivity, resting heart rate, and blood pressure before and after the Rebalance© Program (mean ± SD).

Parameter		Pre-REB	Post-REB	Change (%)	*p*	Cohen’s *d*
Sleep quality (SSQ)^††^	G-con	18.8 ± 5.0	19.4 ± 3.7	3.2	0.74	0.14
	G-nor	19.5 ± 2.5	22.5 ± 3.3	15.4	0.061	1.02
	G-hyp	16.1 ± 3.1	22.9 ± 3.1	42.2	<0.01 0.001	2.19
Sleep reactivity (FIRST)^†^	G-con	23.8 ± 5.3	25.2 ± 5.7	5.8	0.53	0.25
	G-nor	26.6 ± 6.9	26.6 ± 5.7	0.0	1.00	0.00
	G-hyp	28.1 ± 2.5	24.3 ± 5.5	−13.5	0.062	0.89
Resting heart rate (bpm)^††^	G-con	75.2 ± 10.7	73.1 ± 11.4	−2.8	0.63	0.19
	G-nor	72.4 ± 8.7	71.5 ± 8.7	−1.2	0.77	0.10
	G-hyp	81.1 ± 10.8^∆∆^^,^^°^	68.1 ± 9.2	−16.0	<0.01 0.003	1.30
Systolic blood pressure (mmHg)^††^	G-con	129.6 ± 17.0	127.8 ± 16.2	−1.4	0.68	0.11
	G-nor	120.8 ± 9.3	117.7 ± 7.5	−2.6	0.31	0.37
	G-hyp	150.2 ± 8.3^∆∆^^,^^°°^	130.8 ± 14.1°°	−12.9	<0.01 0.0003	1.68
Diastolic blood pressure (mmHg)^†^	G-con	75.5 ± 5.1	79.2 ± 9.3	4.9	0.98	0.49
	G-nor	74.6 ± 7.1	71.1 ± 7.0	−4.7	0.18	0.50
	G-hyp	86.4 ± 9.4^∆∆^^,^^°°^	77.9 ± 10.0°	−9.8	0.036	0.88

Pre-REB, prior to Rebalance© Program, Post-REB, following Rebalance© Program. G-con, control group; G-nor, normotensive group; G-hyp, hypotensive group. SSQ, Spiegel Sleep Quality questionnaire; FIRST, Ford Insomnia Response to Stress Test. The criteria to interpret the magnitude of the effect size were as follows: >0.2 small, >0.5 moderate, >0.8 large, and > 1.3 very large.

† Interaction effect (*p* < 0.05).

†† Interaction effect (*p* < 0.01).

∆∆ Significantly different from G-con (*p* < 0.01).

° Significantly different from G-nor (*p* < 0.05).

°° Significantly different from G-nor (*p* < 0.01).

**Table 3:** Sleep actigraphy data at selected time points throughout the Rebalance© Program (mean ± SD).

Parameter		Baseline	REB-3	REB-6	REB-9
Time in bed (h:min)	G-con	7:42 ± 0:24	7:48 ± 0:30	7:54 ± 0:30	7:48 ± 0:30
	G-nor	7:30 ± 1:06	7:36 ± 1:00	7:42 ± 0:42	7:30 ± 0:42
	G-hyp	7:36 ± 0:48	7:36 ± 0:24	7:36 ± 1:00	7:12 ± 0:48
Assumed sleep time (h:min)	G-con	7:18 ± 0:30	7:24 ± 0:36	7:30 ± 0:24	7:30 ± 0:36
	G-nor	7:06 ± 1:06	7:18 ± 1:00	7:18 ± 0:42	7:06 ± 0:42
	G-hyp	7:12 ± 0:48	7:12 ± 0:30	7:12 ± 1:00	6:48 ± 0:48
Actual sleep time (h:min)	G-con	6:06 ± 0:36	6:12 ± 0:30	6:18 ± 0:24	6:12 ± 0:30
	G-nor	6:00 ± 1:00	6:12 ± 0:54	6:12 ± 0:42	6:06 ± 0:42
	G-hyp	6:06 ± 0:48	6:12 ± 0:36	6:06 ± 0:48	5:54 ± 0:48
Sleep efficiency (%)	G-con	83.7 ± 4.3	83.1 ± 2.9	83.9 ± 3.4	83.4 ± 4.6
	G-nor	85.4 ± 3.6	84.6 ± 3.2	85.1 ± 4.6	85.6 ± 4.0
	G-hyp	84. 3 ± 5.6	85.1 ± 4.4	84.7 ± 4.8	86.5 ± 3.8^*^^,^^§^
Immobile time (%)^†^	G-con	84.1 ± 4.1	83.7 ± 2.5	84.8 ± 2.9	84.3 ± 3.9
	G-nor	85.2 ± 3.0	85.4 ± 2.3	85.5 ± 2.9	86.1 ± 2.5
	G-hyp	85.9 ± 4.6	86.6 ± 3.3	85.9 ± 4.0	87.5 ± 2.9
Fragmentation index^†^	G-con	34.6 ± 10.0	33.5 ± 8.9	33.0 ± 8.9	34.1 ± 9.8
	G-nor	32.0 ± 6.1	31.6 ± 3.6	32.3 ± 6.8	29.0 ± 6.6^*^^,^^§^
	G-hyp	30.3 ± 8.3	28.5 ± 7.1	32.7 ± 5.9	26.6 ± 7.0^*^^,^^§§^

*Baseline, 3-day data collection period prior to Rebalance© session 1; REB-3, −6, −9, 3-day data collection period following Rebalance© session number 3, 6, and 9, respectively. G-con, control group; G-nor, normotensive group; G-hyp, hypotensive group.*

† Significant time–group interaction (*p* < 0.05).

* Significantly different from Baseline (*p* < 0.05).

§ Significantly different from previous measure (*p* < 0.05).

§§ Significantly different from previous measure (*p* < 0.01).

### Resting Heart Rate and Blood Pressure

Resting heart rate and blood pressure values assessed before the Rebalance© Program were significantly higher in the G-hyp group compared to the G-con and G-nor groups (Table 2). A significant time–group interaction was reported in resting heart rate (*p* < 0.01), systolic blood pressure (*p* < 0.01), and diastolic blood pressure (*p* < 0.05). After the Rebalance© Program, a significant decrease in blood pressure combined with a significant decrease in resting heart rate was observed in the G-hyp group, with no change for the G-nor and G-con groups.

### Heart Rate Variability

**Figure 4:**
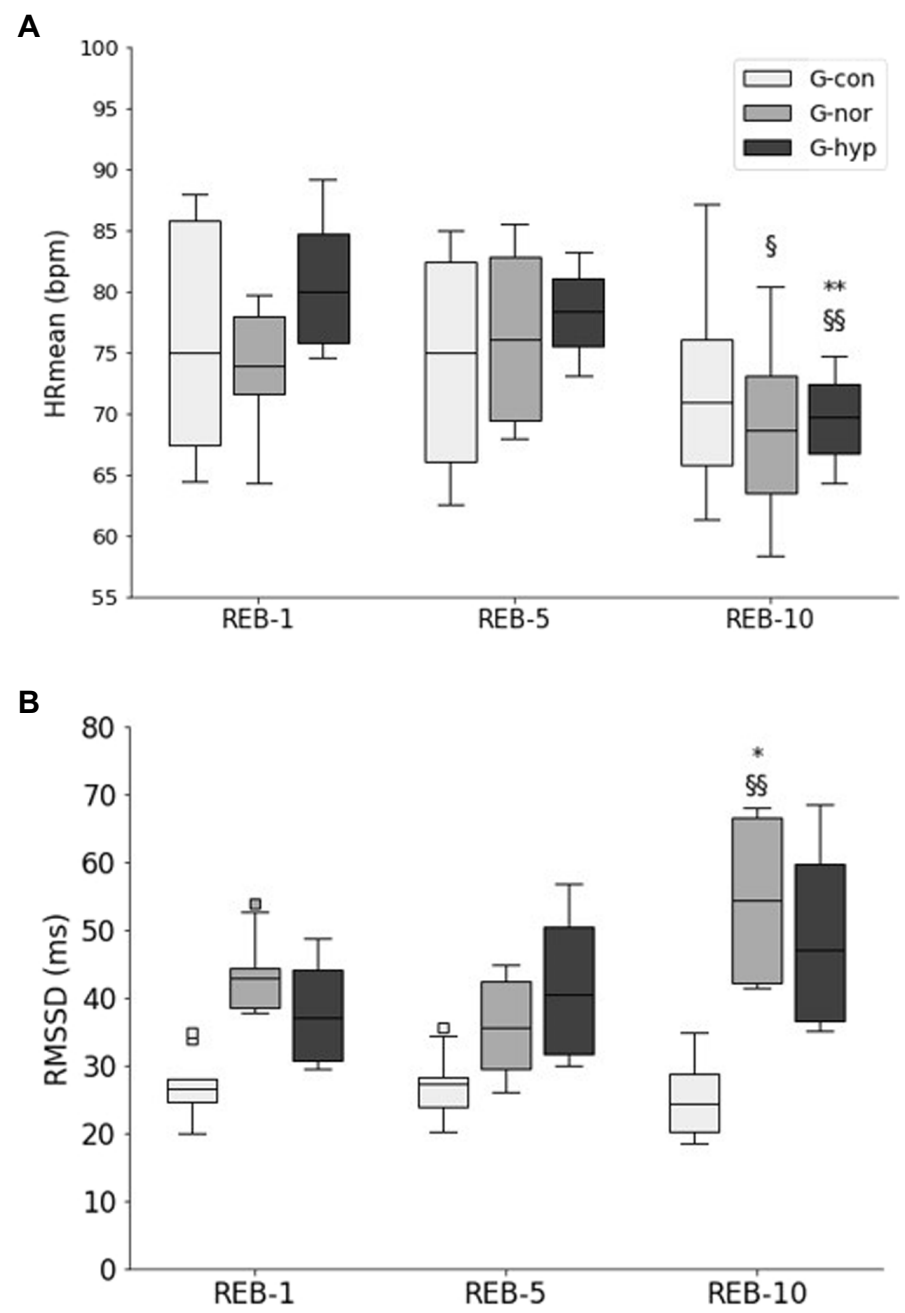
Changes in HRmean **(A)**, RMSSD **(B)** throughout the Rebalance© Program. G-con, control group; G-nor, normotensive group; G-hyp, hypotensive group. REB-1, −5, −10, Rebalance© session number 1, 5, and 10, respectively. HRmean, average heart rate during HRV measurement; HRV, heart rate variability; RMSSD, root-mean square difference of successive normal heart beats. Box plots represent median interquartile range (IQR, Q25–Q75), and error bars are maximal and minimal observations within 1.5 × IQR. Squares represent maximum and minimum observations above or below 1.5 × IQR. * and **Significantly different from REB-1 (*p* < 0.05 and *p* < 0.01, respectively); ^§^ and ^§§^Significantly different from REB-5 (*p* < 0.05 and *p* < 0.01, respectively).

A significant time–group interaction was reported in HRmean (*p* < 0.05) and RMSSD (*p* < 0.01). In response to the Rebalance© Program, HRmean decreased significantly between REB-5 and REB-10 for both experimental groups (*p* < 0.05; Figure 4A), with respective changes of −7.5 ± 11.3 bpm and − 8.7 ± 0.1 bpm for the G-nor and G-hyp groups. In addition, significant changes in RMSSD were only reported in the G-nor group (Figure 4B), with an increase of 56.8% ± 42.5 between REB-5 and REB-10 (*p* < 0.01). For the G-con group, no significant change in HRmean and RMSSD was observed.

### Endocrine Analysis

**Figure 5:**
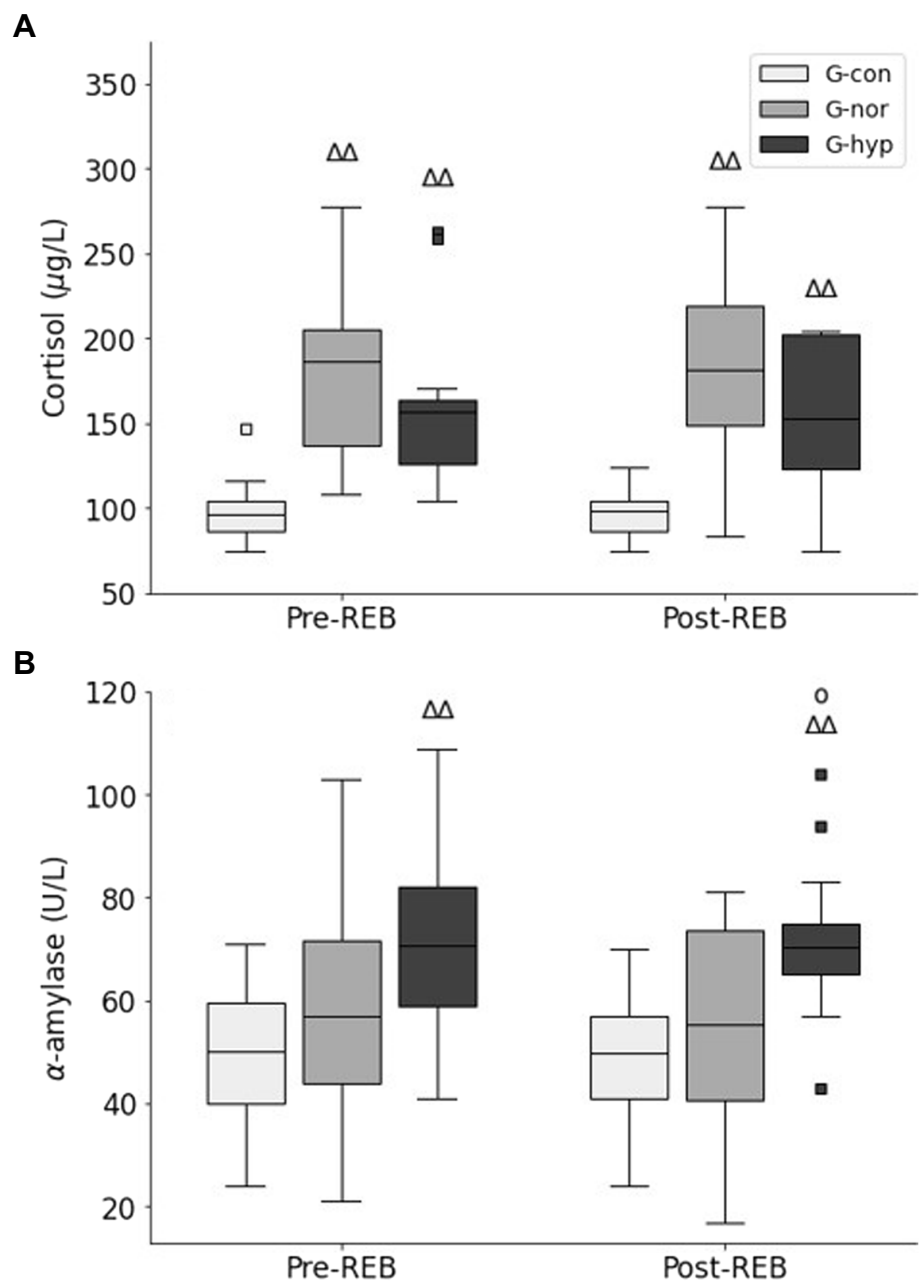
Changes in cortisol **(A)** and α-amylase **(B)** concentrations measured before and after the Rebalance© Program. G-con, control group; G-nor, normotensive group; G-hyp, hypotensive group. Pre-REB, prior to Rebalance© Program; Post-REB, following Rebalance© Program. Box plots represent median interquartile range (IQR, Q25–Q75), and error bars are maximal and minimal observations within 1.5 × IQR. Squares represent maximum and minimum observations above or below 1.5 × IQR. ^∆∆^Significantly different from G-con (*p* < 0.01); °Significantly different from G-nor (*p* < 0.05).

Before and after the Rebalance© Program, cortisol blood concentrations were significantly higher in the G-nor and G-hyp groups compared with the G-con group (*p* < 0.01; Figure 5A), with no difference between the two experimental groups. Higher α-amylase blood concentrations in the G-hyp group were also observed compared with the G-con and G-nor groups in Pre-REB (*p* < 0.01 and *p* = 0.07, respectively) and Post-REB (*p* < 0.01 and *p* < 0.05, respectively). No significant time effect and time–group interaction was reported in blood concentrations values.

## Discussion

This study aimed to determine the effect of a MBI on stress-related parameters in nurses during the COVID-19 pandemic. The main finding of this study was that following 10, 30-min sessions of neuro-meditation using the Rebalance© Impulse the perceived sleep quality of all participants improved, and blood pressure was significantly reduced in those where it had been previously elevated. In participants that completed the MBI, cardiac regulation was also improved, as observed by a decrease in resting heart rate for the G-hyp group and an increase in HRV for the G-nor group. Combined, these results provide support for the implementation of neuro-meditation sessions to improve the health of nurses during periods of increased work stress.

While the effectiveness of MBI for nurses has been previously assessed with promising results, most studies have examined stress reduction *via* psychometric questionnaires and inventories (van der Riet et al., 2018; Yang et al., 2018). In comparison, the current investigation presents a more mechanistic view of the effectiveness of MBI through the measurement of physiological based markers of stress. This provides an opportunity to add to the existing knowledge of the psychosocial and perceptual benefits of MBI by also quantifying the pathophysiological responses. The primary measures used to quantify the impact of increased work-related stress in the present study were sleep parameters, blood pressure, and heart rate (resting and HRV). These assessments were chosen to capture the outcome of the complex interplay between stress, burnout, sleep, and the sympathetic nervous system.

According to the FIRST scores, all participants in the study reported elevated levels of sleep reactivity and were at high risk of developing insomnia (FIRST score > 18; Kalmbach et al., 2016). This impaired sleep quality was also reflected in SSQ scores below 24 in all participants. A key finding was that the Rebalance© Program improved sleep quality by increasing sleep efficiency and reducing the amount of fragmented sleep. This agrees with a meta-analysis that reported a moderate strength of evidence that MBI improved sleep quality in those with an existing sleep disturbance compared to non-specific active controls (Rusch et al., 2019). Of relevance is that the enhanced sleep quality (perceived and SSQ) following the Rebalance© program was associated with improved sympathetic control and autonomic regulation without any change in sleep pattern (i.e., time in bed and actual sleep time). Using a subjective (i.e., FIRST and SSQ) and objective (i.e., actigraphy) approach to sleep evaluation, allowed the detection of a dissociation between perceived and actual sleep efficiency. This is of clinical significance as it suggests that cognitive behavioral techniques are an important aspect for the treatment of sleep disorders. It also suggests that stress-related parameters can be improved by MBI without the need for a longer sleep duration or an altered bedtime. This is particularly important for nursing staff with demanding workloads as they may not be able to allocate additional time to sleep within their schedule.

Despite large effect sizes for the G-nor group, FIRST and SSQ scores were only improved in the G-hyp following the MBI. Previous studies have also provided preliminary, yet mixed evidence for the use of MBIs for sleep disturbances in adults (Gross et al., 2011; Rusch et al., 2019). This discrepancy may be due to sleep typically being assessed as a secondary outcome to a primary disease state, leading to the findings being confounded by changes in the primary ailment (Andersen et al., 2013). In the current investigation, the workload stress due to the pandemic for the nurses was consistent, decreasing the potential impact of external variables and providing an opportunity to better assess the impact of the MBI on sleep parameters. This suggests that there may have been other circumstances or individual differences that reduced the effectiveness of the Rebalance© Program in the G-nor group. Conversely, it is possible that the participants in the G-hyp group were experiencing an elevated response to the work-related stress, as reflected by their elevated blood pressure at the beginning of the intervention. This finding demonstrates how it can be difficult to ascertain which specific influences are clinically important for parameters associated with stress, as lifestyle factors are often inter-related, and the stress response can be highly individual.

There is accumulating evidence that low sleep efficiency (<85%; Javaheri et al., 2008) and short sleep duration (Sun et al., 2016) are positively associated with the likelihood of hypertension. These sleep-related increases in blood pressure have previously been linked to increases in sympathetic activity (Lusardi et al., 1996). While not a causal relationship, the improved sleep quality following the MBI occurred with a reduction of both SBP and DBP in the G-hyp group. This decrease in blood pressure was of clinical importance as the participants went from being considered as stage 2 hypertensive to having a normal blood pressure (Carey et al., 2018). Moreover, the reduction in SBP observed in G-hyp after completing the Rebalance© Program exceeded the average decrease in SBP (5.37 mmHg, *Z* = −3.66, *p* < 0.01) reported in a meta-analysis of pooled data of other randomized controlled trials investigating the effectiveness of MBI (Pascoe et al., 2017). It was to be expected that the MBI would not further reduce the blood pressure of the G-nor group. These findings demonstrate that MBI can promote lower blood pressure levels and reduce the risk of cardiovascular and cerebrovascular morbidity and mortality for those under increased work-related stress (Carey et al., 2018).

Alongside reduced blood pressure, reduced sympathetic activity can also be reflected by a lower resting heart rate and increased HRV (Fatt et al., 2020), as observed in the present investigation following the Rebalance© Program. Previous studies have also identified through HRV measurement that autonomic disturbances during sleep are central contributors to poor sleep quality and a reduced sense of wellbeing (Fatt et al., 2020). Indeed, a decrease in sympathetic drive may be a key factor linking the benefits of MBI with improved sleep quality and cardiac regulation. Elevated cortisol and α-amylase concentrations may also reflect disturbances within the autonomic nervous system (Cozma et al., 2017). However, in the present study, no change in cortisol or α-amylase was found following the Rebalance© Program. This finding fits within the context of the current MBI research, where the effect of mindful meditation on endocrine measures has produced variable results (Pascoe et al., 2017). For example, a reduced salivary α-amylase, but not cortisol was observed following a MBI in cancer survivors with a sleep disturbance (Lipschitz et al., 2013).

Electroencephalogram (EEG) studies suggest that increased alpha activity represents a calm mind and is a precursor for entering a meditative state (Yang et al., 2018). Theta activity is associated with being in a meditative state (Pasquini et al., 2015) and reflects a high level of attentional engagement (Baijal and Srinivasan, 2010). When individuals experience greater theta activity, increased parasympathetic activity and sympathetic activity suppression have been observed (Yang et al., 2018). To further understand the underlying mechanisms to this response, neuroimaging was conducted prior to and following a 3-day intensive MBI in 35 high stress unemployed males and females (Taren et al., 2015). Compared to a matched control (*n* = 41) who undertook a placebo relaxation program, those that participated in the MBI displayed reductions in right amygdala resting state functional connectivity. The amygdala plays a central role in orchestrating the body’s physiological response to a perceived stressor. This finding provides one potential mechanism to explain the observed reductions in stress-related parameters that occur with MBI (Andersen et al., 2013; Creswell, 2017; Pascoe et al., 2017). Those practiced in mindfulness have an enhanced ability to process negative emotions which blunts the sympathetic response (Kadziolka et al., 2016). Consequently, when challenged with a stressor, MBI can reduce the impact of stress-related responses, such as disrupted sleep that are related to autonomic dysregulation (Meerlo et al., 2008). Evidence suggests that neuro-meditation using the Rebalance© Impulse significantly increases the time spent in the theta–alpha oscillation power spectrum compared to a matched control (Cheron et al., 2022). While speculative, this observation suggests that the improved sleep and cardiac regulation reported in the present study may be due to the Rebalance© Impulse enabling the participants to experience a greater amount of theta activity.

Like other MBIs, the Rebalance© Impulse provides a potential non-pharmacological treatment for those under increased stress. Sleep disorders are often treated with pharmacotherapy (Moloney et al., 2011). However, these treatments may only provide temporary remediation of sleep disturbance; the benefits frequently diminish after drug discontinuation; and there is a risk of residual daytime effects and dependency syndrome (Morin et al., 1999). These limitations highlight the need for alternate treatments and the current findings support the use of MBI as a scalable community-accessible intervention that can improve sleep in adults with moderate sleep disturbances. Similarly, while lifestyle changes, such as weight loss, reducing dietary sodium intake, and increased physical activity, may assist in lowering blood pressure, medication is commonly prescribed to treat hypertension (Carey et al., 2018). Again, evidence from the current study and previous investigations support the use of MBI as a promising non-pharmacological approach to lowering blood pressure. For example, following an eight-week, randomized controlled trial for those with stage 1 hypertension, the MBI participants (*n* = 20) were compared to a matched control (*n* = 16) who completed health education talks. The MBI participants experienced a clinically significant improvement in SBP that remained decreased for up to 20 weeks following the intervention (Ponte Marquez et al., 2019). Collectively, these findings advocate for the use of MBI to reduce stress-related parameters and improve wellbeing without the need for pharmacological interventions.

Benefits of MBI are typically observed when participants complete both dedicated sessions and daily practice (Smith, 2014; van der Riet et al., 2018). However, a dose–response between time spent meditating and improved sleep quality is yet to be established (Rusch et al., 2019). This may be due to the difficulty assessing the actual time spent in a mindful meditative state, compared to when the mind wanders (Davidson and Kaszniak, 2015). The reduction of stress-related parameters occurred in the present study following a relatively short MBI intervention. This supports the notion that the audio and light guided neuro-meditation using the Rebalance© Impulse is a time effective practice that can maximize the time spent in a mindful meditative state. However, it should be noted that other forms of neurofeedback MBI have not provided any additional benefit beyond the more traditional, focused attention mindful meditation practice (Polich et al., 2020). The current findings also highlight that comparable improvements in stress-related parameters can be achieved with neuro-meditation without the need for daily practice. This is of particular relevance for health care workers, considering it has previously been identified that at home mindfulness practice would be difficult for this population to implement and maintain (Foureur et al., 2013).

There are several limitations to the present investigation that should be acknowledged. An active treatment comparison group was not used and participants in the treatment groups were not blinded to the purpose of the intervention. This creates a potential for the benefits of the Rebalance© Program to be attributed to a placebo effect. Also, the age of the participants was heterogeneous and obese participants were not excluded. In addition, including participants with elevated blood pressure in the control group would have provided a direct comparison for the effectiveness of MBI on reducing blood pressure during periods of increased work-related stress. It is also recognized that a relatively small sample size was used. It is also unknown whether the acute changes after the neuro-meditation sessions were maintained as a follow-up at 6 and 12 months was not completed.

## Conclusion

Neuro-meditation provided an effective, non-pharmacological treatment to combat increases in work-related stress symptoms in nurses during the COVID-19 pandemic. While future studies are required to fully elucidate the underlying mechanisms, initial evidence suggests that mindful meditation assists by reducing sympathetic activity, as demonstrated through enhanced sleep and the re-establishment autonomic control. These benefits were observed in participants who were previously untrained in meditative practices. This suggests that compared to other MBI, which often require a greater time commitment and training, the guided neuro-meditation and synchromotherapy accelerated the participants’ ability to achieve the desired meditative state. Collectively, these results support the use of neuro-meditation to promote health and wellbeing in nurses.

## Data Availability Statement

The raw data supporting the conclusions of this article will be made available by the authors, without undue reservation.

## Ethics Statement

The studies involving human participants were reviewed and approved by the University of Technology, Sydney (ETH21-6116). The patients/participants provided their written informed consent to participate in this study.

## Author Contributions

CH: conceptualization, funding acquisition, methodology, research design, project administration, and writing. XN, AD, and FD: investigation and formal analysis. YR: resources, review, and editing. KS: writing and editing. All authors contributed to the article and approved the submitted version.

## Funding

Financial support was provided to compensate the participants of the study and for the biochemical analyses. This study was conducted after the first lockdown due to COVID-19 in solidarity with the nursing staff.

## Conflict of Interest

The authors declare that the research was conducted in the absence of any commercial or financial relationships that could be construed as a potential conflict of interest.

## Publisher’s Note

All claims expressed in this article are solely those of the authors and do not necessarily represent those of their affiliated organizations, or those of the publisher, the editors and the reviewers. Any product that may be evaluated in this article, or claim that may be made by its manufacturer, is not guaranteed or endorsed by the publisher.
